# Hyper-Immune Bovine Milk as an Immunological and Nutritional Supplement for COVID-19

**DOI:** 10.3389/fnut.2022.868964

**Published:** 2022-06-21

**Authors:** Hassan Nili, Majid Bouzari, Hamid Reza Attaran, Nader Ghalegolab, Mohammad Rabani, Ahmad Mahmoudian

**Affiliations:** ^1^Virology Research Center, Faculty of Biological Sciences and Technology, University of Isfahan, Isfahan, Iran; ^2^Zeitoon Isfahan Vaccine Innovators Company, Isfahan Sciences and Technology Town, Isfahan, Iran; ^3^Department of Cellular and Molecular Biology and Microbiology, Faculty of Biological Sciences and Technology, University of Isfahan, Isfahan, Iran; ^4^Razi Serum and Vaccine Research Institute, Agricultural Research, Education and Extension (AREEO), Shiraz, Iran; ^5^Department of Community Medicine, School of Medicine, Isfahan University of Medical Science, Isfahan, Iran

**Keywords:** hyper-immune, bovine milk, nutritional, supplement, COVID-19, neutralizing, antibodies

## Abstract

Many different strategies have been used to fight against the Coronavirus disease (COVID-19) pandemic as a therapeutics or prophylaxis approaches. However, not enough attention has been paid to general and specific immune factors and nutritional components found in hyper-immunized dairy products. Hyper-immune bovine colostrum (HBC) has been used against many different respiratory and gastrointestinal tracts infections during past decades. An isolated dairy farm was established, and nine mixed Holstein X Simmental dairy cattle in their 6–7 months of gestation period were chosen for hyper-immunization with inactivated Severe acute respiratory syndrome corona virus-2 (SARS-CoV-2). For this, six cows were inoculated with 2 ml of 10^9.4^/ml (TCID_50_) of the virus. As a control group, three cows were inoculated with the carrier without virus. Specific IgG level against the SARS-CoV-2 was measured before and after immunization in the sera, and in the colostrum and milk following parturition in hyper-immunized cows using indirect Enzyme-linked immunosorbent assay (ELISA). Neutralizing antibodies in the serum and colostrum was measured by a quantitative ELISA. The safety of the product was determined in40 healthy volunteers aged between 18–65 years old (13 females and 27 males) in the phase 1 clinical trial (https://www.irct.ir/trial/51259). No adverse effects were observed in the experimental cows. A very high level of IgG was observed in the first colostrum that sharply decreased in the following 7 days in the milk. The titer of specific neutralizing antibody in the colostrum samples was 69 times higher than the sera. No adverse effects and clinical complications were reported by the authorized ethics committee, and an official certificate on the safety of the product was issued. Beside other strategies, this approach could be used for large-scale and low-cost production of immune components to be used as a nutritional supplement to confront current SARS-CoV-2 and future pandemics.

**Clinical Trial Registration:**

[https://www.irct.ir/trial/51259].

## Introduction

The emergence of severe acute respiratory syndrome coronavirus 2 (SARS-CoV-2) causing severe human respiratory infection (COVID-19) has become more than a global health crisis (1, 2). It has devastating effects on all aspects of life, from increasing family violence and abuses to the catastrophic effects on the world economy (3). In January 2020, the World Health Organization announced it as a public health emergency with international concern (4). Many scientists in different countries are engaged in intensive research work to solve the issue. Regardless of the availability of specific vaccines and antiviral drugs, COVID-19 continues to have a serious long term impact on human health around the world (5). Although much attention and debates have been focused on the efficacy of different vaccines against COVID-19, not much attention has been paid to nutritional components of dairies which might have immunological implications on the overall human immune response to COVID-19. In this regard, dairy products of hyper-immunized (creating an immune state greater than normal) dairy cows not only will provide massive specific immune components to the consumers, very high biological values of milk and colostrum, but can also enhance the host immune response to the infection. Considering the fact that people, especially children and elderly, in many undeveloped countries, are suffering from a poor diet with low protein and vitamin contents (6). Milk contains essential nutrients like high-quality protein, calcium, and potent antioxidants like vitamins A, D, and more (7). Therefore, using hyper-immune bovine milk, especially in developing countries, is a dual-purpose strategy to fight both COVID-19 viral infection and malnutrition in mothers, infants, children, adolescents, and the elderly. In this regards, the most potent vaccines might not work properly when there is a deficiency of essential nutrients (8).

Considering this, production of large amounts of specific antibodies in animal models such as dairy cattle could be used as an alternative approach against circulating pathogens during pandemics, especially in immunocompromised patients (9). By hyper-immunization of pregnant dairy cows in the late gestation period using specific antigens, the concentration of specific immunoglobulins (Igs) such as IgG in the sera is increased (10, 11). Antibody level, especially IgG1, is reduced in the bloodstream 2–3 weeks before parturition and actively transported through a receptor-mediated mechanism to the lacteal secretions following parturition (12). The total amount of IgG1 obtained from each lactation could be as high as 500 g (12, 13). Oral hyper-immune bovine colostrum (HBC) and milk not only can increase the mucosal immunity in the oral cavity, pharynx, and upper respiratory tract of humans, even could have immunomodulatory effects on the host immune system (14). IgG is one of the main components of immune activity found in milk and colostrum, which can bind to many gastrointestinal and respiratory pathogens that infect humans such as cryptosporidium, *Shigella*, rotavirus, respiratory syncytial virus, human immunodeficiency virus, influenza virus, enterotoxigenic *Escherichia coli*, and *Clostridium difficile* and supports the cross-species activity of bovine and human IgG (14–22).

Igs in breast milk are IgA, IgG1, IgG2, and IgM. IgG1 is the main Ig in cow’s milk, and colostrum, while the concentration of IgM, IgA, and IgG2 are lower (23). The concentration of IgG1 in colostrum is 100 times higher than in milk (10). Besides specific antibodies, bovine colostrum contains many essential nutrients and bioactive components, including growth factors, lactoperoxidase, lysozyme, lactoferrin, cytokines, nucleosides, vitamins, peptides, and oligosaccharides. These components are highly related to human health. For example, vitamin D level which is found in higher concentration in bovine colostrum than in ordinary milk has been shown to be associated with severity of COVID-19 illness (24). IgG from unimmunized cattle can interact with different types of pathogens, including viruses (14).

In relation to immune-modulatory effects of bovine colostrum, it is shown that bovine colostrum increased the proportion of CD8 + T-cells after virus attack in mice (14). Bovine colostrum-derived IgG can inhibit the NF-κB signaling pathway and inhibit the production of proinflammatory cytokines in intestinal cells (25). Hyper-immune bovine IgG can directly bind to pathogenic organisms, including viruses, and prevent adhering to intestinal epithelial cells (20, 26, 27). It has been reported that bovine colostrum-derived IgG is resistant to proteolysis, which supports the view that colostrum contains trypsin inhibitors, which can promote these antibodies to survive throughout the gastrointestinal tract (28, 29).

Therefore, considering previous work on prophylactic and therapeutic effects of bovine colostrum-derived immunoglobulins on different infectious organisms, the same approach was used against COVID-19 infection (14, 16, 18, 21–23, 28, 30–32). This research focuses on the production and safety of hyper-immune milk or colostrum collected from cows vaccinated with SARS-CoV-2 in healthy individuals. By this approach, large-scale and low-cost production of immune components can be achieved to confront pandemics such as SARS-CoV-2.

## Materials and Methods

### Bovines

Nine mixed Holstein X Simmental in their 6–7 months of gestation period aged 3–4 years were chosen for hyper-immunization with inactivated SARS-CoV-2. Their health status was examined by laboratory check outs and clinical examination. The stage of gestation was examined by palpation and sonography by a dairy farm veterinary specialist. An isolated and very well-protected dairy farm was selected and equipped for the experiment in the Zardanjan area in the East of Isfahan-Iran. Animals were kept under close daily observation for adaptation to the new environment. An experienced animal husbandry engineer was employed to supervise dairy cows during the experiment. A special diet for the dry period was purchased from the Vahdat company, Isfahan. One month after arriving, the cows were divided into two treatment (*n* = 6) and control (received vaccine ingredients without the virus) (*n* = 3) groups. Animal experimentation was approved by the ethics committee of the Isfahan University of Medical Sciences (IR.MUI.MED.REC.1399.1029).

### Virus Preparation and Inactivation

The virus was produced according to the protocol of influenza vaccine production, and FDA approved adjuvant (Montanide oil) was used as well. Briefly, the virus was isolated from the nasopharynx samples taken from COVID-19 positive patients, cultured on the WHO Vero cell line according to Kim et al. (33). The presence and purity of SARS-CoV-2 were checked by RT-PCR, Nano-Sensor (34), and serum neutralizing tests. Each ml of the preparation contained 10^9.4^ (TCID_50_) of SARS-CoV-2. For viral inactivation formaldehyde was used. Inactivation was tested in mouse, rat and Syrian hamsters in groups of five (35, 36).

Bacterial culture was performed on nutrient broth, blood agar, tryptic soy broth, thioglycolate broth, PPLO broth media in aerobic and anaerobic conditions. Also, sabourauddextrose agar was used to test the presence of fungi.

### Virus Inoculation

Six dairy cows were inoculated with 2 ml of SARS-CoV-2 and two boosters in 2 weeks intervalsintramuscularly in the thigh muscle as a treatment group before parturition. Three cows in the control group were inoculated with all the ingredients except for the virus.

### Clinical Observation

Before and after virus inoculation, pregnant cows were closely monitored daily for any changes in behavior such as occasional systemic shock, itching, swelling, or any adverse effects in the vaccination site in the thigh muscle. Also, they were monitored for any change in water and feed consumption, restlessness, and increase in body temperature.

### Blood Samples

All laboratory experiments were conducted in the Virology Research Center of the University of Isfahan in conjunction with Zeitoon Isfahan Vaccine Innovators Company facilities. Blood samples were collected at weekly intervals from the milk vein and kept at room temperature for 30 min to coagulate, and then centrifuged at 2,500 g for 15 min at room temperature. Sera were removed and stored in aliquots at –20°C before use.

### Preparation of Colostrum and Milk

HBCs were collected immediately after parturition and quickly pasteurized at 60°C for at least 60 min [Low-temperature long time (LTLT)]. After pasteurization, the colostrum temperature was brought to 4°C, and transferred to the laboratory to keep at –20°C, until use. The same was performed for milk preparation collected at 3, 5, and 7 days after parturition. Frozen colostrum samples were thawed, centrifuged at 11,000 g for 15–30 min at 4°C. After removal of fat, the supernatants were collected.

### Severe Acute Respiratory Syndrome Coronavirus 2 Antibody In-Direct Enzyme-Linked Immunosorbent Assays

The milk, sera and colostrum supernatants were diluted 1/100, 1/200 1/400, and 1/1,000 using kit diluent, and specific IgG was measured using an in-direct ELISA. ELISA was performed according to ELISA Kit SARS-CoV-2 IgG (Pishtazteb, Iran) protocol with some modification. The diluted test samples (100 μl) were added to the wells pre-coated with N antigen of the virus at 37°C for 30 min. After washing of the plates, horseradish peroxidase-conjugated anti-bovine IgG antibody (Sigma) (100 μl) was used as the secondary antibody and incubated at 37°C for 30 min and after washing, 100 μl of chromatogen-substrate solution (tetramethyl bezidin) was added, then 100 μl of the stop solution was added and test was read at 450 nm wavelength. Duplicate positive controls containing anti-SARS-CoV-2 IgG and negative controls (without IgG antibody) were included in the test.

#### Neutralizing Antibodies

Selected serum samples before and after vaccination, and colostrum supernatants were used to measure neutralizing antibodies. A quantitative competitive ELISA kit of PishtazTeb (Tehran, Iran) was used to measure neutralizing antibodies. Six wells were used for different standards (50 μl) (0, 1, 2.5, 5, 10, and 40 μg/ml of neutralizing antibodies) negative and positive control were included according to the manufacturer instructions for preparation of standard curve. Briefly, receptor binding domain (RBD) coated wells were filled with either serum samples, milk or colostrum samples (50 μl). Angiotensin-converting enzyme 2 (ACE2) conjugated with HRP (50 μl were added to the wells simultaneously) were shaken for 15 s and incubated at 37°C for 30 min. Neutralizing antibodies against RBD-SARS-CoV-2 present in serumand colostrum samples were attached to the antigens (RBD) and prevented the conjugate from joining RBD. Following washing, with 30 μl of washing buffer for five times, 100 μl of chromagen (blue dye) was added to the wells and were incubated at room temperature for 15 min, covered to avoid light exposure. There was an opposite correlation between immune complex formation at the bottom of the wells and the intensity of dye color. By adding 100 μl of the stop buffer, the blue color turned to yellow, and the best absorbance was detectable at 450 nm wavelength. All standards, positive and negative controls, and tests were performed in duplicate.

### Clinical Trial Phase I

Forty healthy volunteers, aged 18–65 years (Table 1), with no background medical complications, were participated in the trial under the supervision of the ethical committee for research projects in biological sciences with an ethical code of IR.MUI.REC.1399.672 of Isfahan University of Medical Sciences and Iranian clinical trials registry No: IRCT20200927048849N1. Each individual signed the consent form before entering phase 1 of the clinical trial. Volunteers were given 150 ml per day of colostrum enriched milk for up to 30 consequent days. Name, national ID numbers, and phone numbers of the participants were given to the ethics committee of Isfahan University of Medical Sciences for further follow-up. After 14 days, their clinical status (necessity of emergency medical service, gastrointestinal disorders, hospitalization, and any medical problem) was assessed through phone interview by expert medical personnels from independent investigation team of the ethics committee.

**TABLE 1:** Age and sex distribution of participants in phase 1 of the clinical trial.

Age groups	Sex	
	Male	Female
18–20	0	2
21–30	4	1
31–40	6	7
41–50	5	1
51–60	8	1
61–65	3	2

## Results

### Virus Inoculation and Inactivation

The isolated virus was adapted to vero cells by several passages reaching to the TCID 50 of 10^9.4^. The presence of the virus was confirmed by RT-PCR (C_*t*_-10) and serum neutralizing test. No virus was detected by RT-qPCR in pharyngeal swabs and blood samples of the mice, rats and Syrian hamsters inoculated with the inactivated virus at least 2 weeks post-inoculation. Furthermore, no clinical signs were observed. No bacterial and fungal contamination were detected in the virus preparation.

### Bovines

#### Clinical Observation

Veterinary and laboratory check outs of the pregnant animals and their fetus did not show any abnormalities before and after vaccination. No changes were observed in the behavior and clinical signs such as body temperature, feed, and water consumption of inoculated pregnant animals. Also, adverse tissue reactions were not detected on the injection site in the thigh muscle after the virus inoculation.

#### Enzyme-Linked Immunosorbent Assay

**FIGURE 1:**
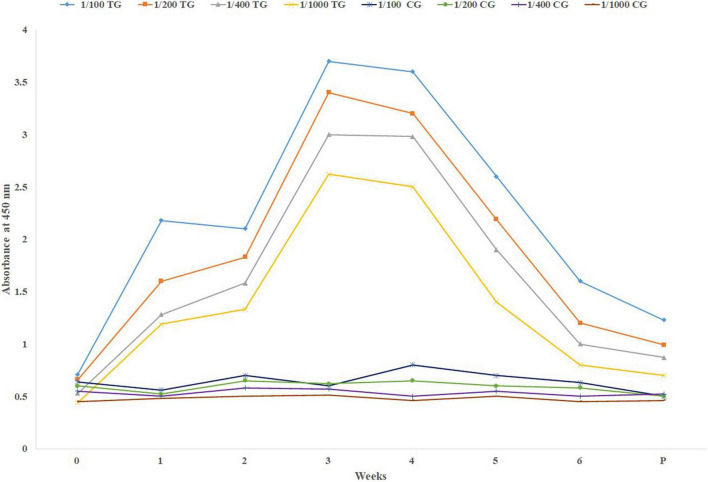
Mean specific IgG level in different sera dilutions of six treatment and three negative control pregnant cows before parturition, using in-direct ELISA test. TG, Treatment group; CG, Control group; Week 0, First virus inoculation; Week 2, First booster; Week 4, Second booster; P, Parturition.

Comparison of dynamism of specific IgG level (mean) among the sera collected before and at parturition is shown in Figure 1. As it is shown, in Figure 1 there is a sharp increase in the specific IgG level against SARS-CoV-2 following the first booster. Just before parturition it was decreased sharply.

**FIGURE 2:**
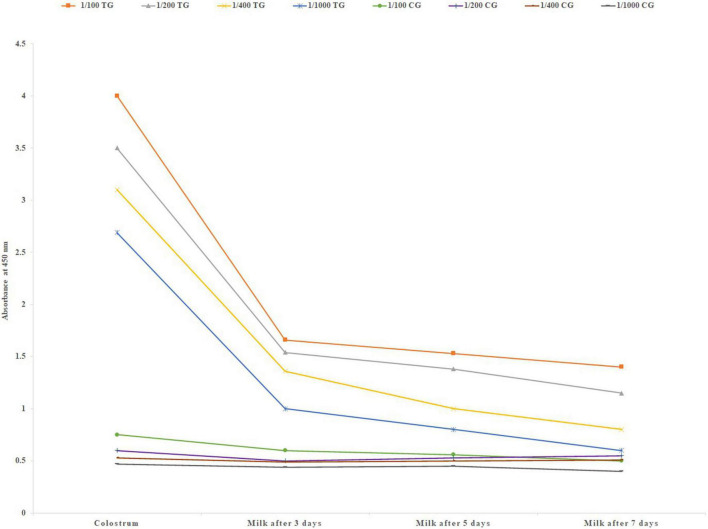
Mean specific IgG level in different colostrum and milk dilutions of six treatment and three negative control pregnant cows after parturition, using in-direct ELISA test. TG, Treatment group; CG, Control group.

Immediately after parturition first colostrum samples were collected. Also milk was collected up to 7 days after parturition. A very high level of IgG was observed in the first colostrum samples that sharply decreased in the following 7 days in the milk (Figure 2). A high level of mean specific IgG was detected in the lowest dilution of the first colostrum (1/1000).

#### Neutralizing Antibody Assay

**FIGURE 3:**
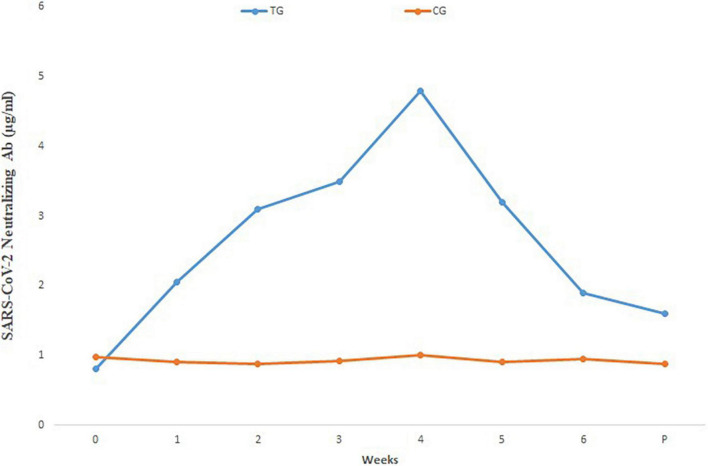
Mean SARS-CoV-2 neutralizing antibody in different sera dilutions of six treatment and three negative control pregnant cows before and at parturition, using ELISA test. TG, Treatment group; CG, Control group; Week 0, First virus inoculation; Week 2, First booster; Week 4, Second booster; P, Parturition.

**FIGURE 4:**
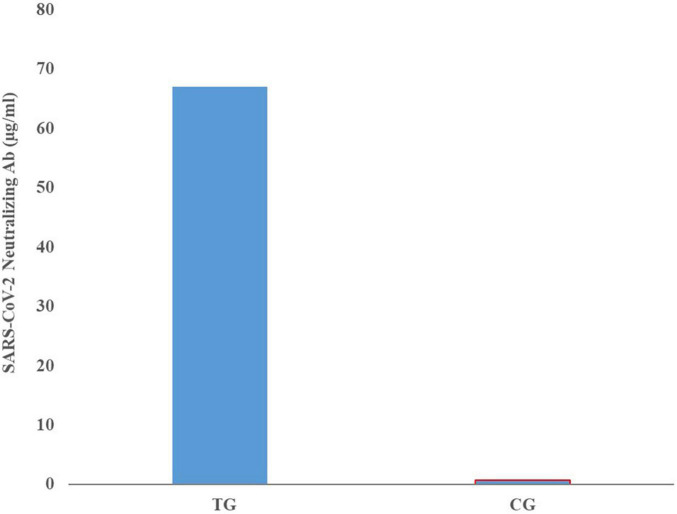
Mean neutralizing antibody titers in the colostrum samples of inoculated and control groups.

Comparison of mean neutralizing antibody titers in the sera of pregnant inoculated before and at parturition in treatment and control group cows are shown in Figure 3. Comparison of mean of neutralizing antibodies in the colostrum samples of treatment and control cows is shown in Figure 4. The titer of the neutralizing antibodies in treatment cows was 89 times higher than the control.

##### Clinical Trial Phase I

No adverse effects and clinical complications were reported by the ethics committee and an official certificate on the safety of the product was issued.

## Discussion

During exposure of the body to infectious organisms, an additional burden is imposed on the immune system. Therefore, the balance of nutrients intake could help infected patients to more efficiently produce appropriate immune components, biochemical molecules and to facilitate specific immune cells to proliferate (37, 38). It is noteworthy to mention that the impact of diet quality on the severity of SARS-CoV-2 infection is getting more attention by the medical community. Therefore, even ordinary bovine milk, which has high biological value, could provide nutrients such as antioxidants, amino acids, vitamins, macro, and micronutrients needed for the immune system to answer extra metabolic demands required to fight pathogenic organisms (39, 40).

Pregnant cows behaved normally following inoculation of inactivated SARS-CoV-2 and did not show any clinical signs and mortalities. There was no abortion, fever or changes infeed and water consumption and tissue reaction in the inoculation sites.

The safety of the product was approved in phase I of the clinical trial during this study.

Although many FDA-approved and investigational antiviral drugs, alone or in combination, are in use during the ongoing SARS-CoV-2 pandemic, none of the clinical trials so far have used bovine colostrum-based immune components against COVID-19 (41). Immunomodulatory effects of HBC have already been studied in different stages of various diseases (10–13, 15–17). The application of bovine Ighas been used in many respiratory and gastrointestinal tracts infections during the last two decades (14, 20–22, 29, 34). For more than 100 years, it has been recognized that milk and colostrum provide passive immunity to a newborn infant *via* the transfer of bioactive factors and Igs (42). The unique physiology of antibody transfer from placenta of mother to neonate in ruminants which does not allow passage of immunoglobulins from dam to fetus, provides all immune components in the colostrum. This phenomenon provides the dairy industry with massive amounts of antibodies, immediately after parturition (12). By hyper immunization of pregnant cows in their late gestation period, we can increase the specificity of immune components available to us after parturition. Perhaps because of this unique function, ruminant neonates are borne without Igs, and 70–80 percent of total protein content in their colostrum are Igs (11, 43).

The results of the current study show that the IgG level in the sera starts to decline 2–3 weeks before parturition, and this is because of active receptor-mediated transfer of the antibody from the blood stream to the mammary glands. These results are in agreement with previous research by Burton et al. (12).

The level of IgG in the bloodstream did not increase after the second booster. This could be due to a limited time period before parturition.

High IgG level obtained even in the lowest dilution of the sera and colostrum, shows that the inactivated virus inoculation method used in this study has been able to induce a proper humoral immune response and the titer of specific neutralizing antibody in the colostrum samples was 69 times higher than the sera and 89 times higher than control colostrum samples.

Phase one of the clinical trial was conducted to determine the safety of hyper-immune bovine milk. Using 150 ml of the product daily for up to 30 consequence days did not have any adverse effects in healthy volunteers aged between 18–65 years.

In addition to currently approved antiviral therapies, passive transfer of immune components through the oral route of dairy products could be an alternative strategy against viruses, including the SARS-CoV-2 (14, 44). Although several new therapeutic strategies are emerging in this desperate time, none have been based on specific bovine-derived immunoglobulins.

Application of immunoglobulins by oral route has no contradiction with vaccination, and US food and drug administration (FDA) has approved the safety of hyperimmune milk on the basis of clinical studies (39, 40). It is proved that orally ingested HBC immunoglobulins are functionally active during passage through the gastrointestinal tract and, along with other components, can prevent upper respiratory and gastrointestinal tracts infections and also lipopolysaccharide-related inflammation. Partial immune exclusion of pathogens happens when milk passes through the nasopharynx, especially if it is kept there for a while before swallowing (22). Besides neutralizing activity of bovine IgG, Fc of bovine IgG can bind to Fc-gamma receptors (FcγRII) of human monocytes, macrophages, natural killer cells, and neutrophils. Attachment of Fc to the FcγRII, along with the killing of the pathogens by phagocytosis, and antibody-dependent cellular toxicity, initiates antigen presentation, which may increase adaptive immunity by enhancing T and B cell responses and production of pathogen-specific IgA antibodies (14). Therefore, it can be hypothesized that oral consumption of BHC against SARS-CoV-2 infection before and during initial infection of the nasopharynx and intestinal tract may be able to reduce the active virions by neutralizing them and also initiate innate and adaptive immune responses at mucosal surfaces of respiratory and gastrointestinal tracts. However, this should be confirmed for SARS-CoV-2 by further studies.

In conclusion, generation of IgG and neutralizing antibodies was successful in the bovine colostrum which is safe for consumption.

## Data Availability Statement

The original contributions presented in the study are included in the article/supplementary material, further inquiries can be directed to the corresponding author/s.

## Ethics Statement

The studies involving human participants were reviewed and approved by the Ethics Committee of the Isfahan University of Medical Sciences. The patients/participants provided their written informed consent to participate in this study. The animal study was reviewed and approved by Ethics Committee of the Isfahan University of Medical Sciences.

## Author Contributions

All authors contributed in this manuscript made substantial contributions to the conception or design of the work, the acquisition, analysis, and interpretation of data, the creation of new software used in the work, drafted the work and revised it critically for important intellectual content, agreed to be accountable for all aspects of the work in ensuring that questions related to the accuracy and integrity of any part of the work were appropriately investigated and resolved.

## Conflict of Interest

HN and MB were employed by the Zeitoon Isfahan Vaccine Innovators Company. The remaining authors declare that the research was conducted in the absence of any commercial or financial relationships that could be construed as a potential conflict of interest.

## Publisher’s Note

All claims expressed in this article are solely those of the authors and do not necessarily represent those of their affiliated organizations, or those of the publisher, the editors and the reviewers. Any product that may be evaluated in this article, or claim that may be made by its manufacturer, is not guaranteed or endorsed by the publisher.

## References

[1] Jawhara S. Could Intravenous immunoglobulin collected from recovered coronavirus patients protect against COVID-19 and strengthen the immune system of new patients? *Int J Mol Sci.* (2020) 21:2272. 10.3390/ijms21072272 32218340 PMC7178250

[2] Lin L Lu L Cao W Li T. Hypothesis for potential pathogenesis of SARS-CoV-2 infection-a review of immune changes in patients with viral pneumonia. *Emerg Microb Infect.* (2020) 9:727–32. 10.1080/22221751.2020.1746199 32196410 PMC7170333

[3] Campbell AM. An increasing risk of family violence during the covid-19 pandemic: strengthening community collaborations to save lives. *Forensic Sci Int Rep.* (2020) 2:100089. 10.1016/j.fsir.2020.100089 PMC7152912 38620174

[4] Sohrabi C Alsafi Z O’Neill N Khan M Kerwan A Al-Jabir A World Health Organization declares global emergency: a review of the 2019 novel coronavirus (COVID-19). *Int J Surg.* (2020) 76:71–6. 32112977 10.1016/j.ijsu.2020.02.034 PMC7105032

[5] Lopez-Leon S Wegman-Ostrosky T Perelman C Sepulveda R Rebolledo PA Cuapio A More than 50 long-term effects of COVID-19: a systematic review and meta-analysis. *Sci Rep.* (2021) 11:16144. 10.1038/s41598-021-95565-8 PMC8352980 34373540

[6] Adesogan AT Havelaar AH McKune SL Eilittä M Dahl GE. Animal source foods: sustainability problem or malnutrition and sustainability solution? Perspective matters. *Glob Food Secur.* (2020) 25:100325.

[7] Chalupa-Krebzdak S Long CJ Bohrer BM. Nutrient density and nutritional value of milk and plant-based milk alternatives. *Int Dairy J.* (2018) 87:84–92.

[8] Calder PC. Nutrition and immunity: lessons for COVID-19. *Eur J Clin Nutr.* (2021) 75:1309–18. 34163017 10.1038/s41430-021-00949-8 PMC8220366

[9] Guzman E Montoya M. Contributions of farm animals to immunology. *Front Vet Sci.* (2018) 5:307. 10.3389/fvets.2018.00307 30574508 PMC6292178

[10] Hurley WL Theil PK. Perspectives on immunoglobulins in colostrum and milk. *Nutrients.* (2011) 3:442–74. 10.3390/nu3040442 22254105 PMC3257684

[11] Butler JE. Immunoglobulin diversity, B-cell and antibody repertoire development in large farm animals. *Rev Sci Tech.* (1998) 17:43–70. 10.20506/rst.17.1.1096 9638800

[12] Burton R Kim S Patel R Scola M Hartman D Tracey D Serum and colostral antibody production in cows immunized with recombinant human tumor necrosis factor. *J Dairy Sci.* (2016) 99:4739–49. 10.3168/jds.2016-10863 27040787

[13] Korhonen HJ Marnila P. 10 – Bovine milk immunoglobulins against microbial human diseases. In: Corredig M editor. *Dairy-Derived Ingredients.* Sawston: Woodhead Publishing (2009). p. 269–89. 10.1016/j.vaccine.2009.04.034

[14] Xu ML Kim HJ Wi GR Kim HJ. The effect of dietary bovine colostrum on respiratory syncytial virus infection and immune responses following the infection in the mouse. *J Microbiol.* (2015) 53:661–6. 10.1007/s12275-015-5353-4 26310306

[15] Weiner C Pan Q Hurtig M Borén T Bostwick E Hammarström L. Passive immunity against human pathogens using bovine antibodies. *Clin Exp Immunol.* (1999) 116:193–205. 10.1046/j.1365-2249.1999.00880.x 10337007 PMC1905285

[16] Ebina T Sato A Umezu K Ishida N Ohyama S Ohizumi A Prevention of rotavirus infection by cow colostrum antibody against human rotaviruses. *Lancet.* (1983) 2:1029–30. 10.1016/s0140-6736(83)91016-4 6138575

[17] Yolken RH Losonsky GA Vonderfecht S Leister F Wee SB. Antibody to human rotavirus in cow’s milk. *N Engl J Med.* (1985) 312:605–10. 10.1056/NEJM198503073121002 2983202

[18] Florén CH Chinenye S Elfstrand L Hagman C Ihse I. ColoPlus, a new product based on bovine colostrum, alleviates HIV-associated diarrhoea. *Scand J Gastroenterol.* (2006) 41:682–6. 10.1080/00365520500380817 16716966

[19] Odong P Angwech P Obol J Florén C. Management of HIV in children using a bovine colostrum-based food product— an observational field study. *World J AIDS.* (2015) 5:100–4.

[20] den Hartog G Jacobino S Bont L Cox L Ulfman LH Leusen JH Specificity and effector functions of human RSV-specific IgG from bovine milk. *PLoS One.* (2014) 9:e112047. 10.1371/journal.pone.0112047 25375837 PMC4222812

[21] Ng WC Wong V Muller B Rawlin G Brown LE. Prevention and treatment of influenza with hyperimmune bovine colostrum antibody. *PLoS One.* (2010) 5:e13622. 10.1371/journal.pone.0013622 21049034 PMC2964324

[22] Kramski M Center RJ Wheatley AK Jacobson JC Alexander MR Rawlin G Hyperimmune bovine colostrum as a low-cost, large-scale source of antibodies with broad neutralizing activity for HIV-1 envelope with potential use in microbicides. *Antimicrob Agents Chemother.* (2012) 56:4310–9. 10.1128/AAC.00453-12 22664963 PMC3421555

[23] Ulfman LH Leusen JHW Savelkoul HFJ Warner JO van Neerven RJJ. Effects of bovine immunoglobulins on immune function, allergy, and infection. *Front Nutr.* (2018) 5:52. 10.3389/fnut.2018.00052 29988421 PMC6024018

[24] Dror AA Morozov N Daoud A Namir Y Yakir O Shachar Y Pre-infection 25-hydroxyvitamin D3 levels and association with severity of COVID-19 illness. *PLoS One.* (2022) 17:e0263069. 10.1371/journal.pone.0263069 35113901 PMC8812897

[25] An MJ Cheon JH Kim SW Park JJ Moon CM Han SY Bovine colostrum inhibits nuclear factor kappaB-mediated proinflammatory cytokine expression in intestinal epithelial cells. *Nutr Res.* (2009) 29:275–80. 10.1016/j.nutres.2009.03.011 19410980

[26] Rump JA Arndt R Arnold A Bendick C Dichtelmüller H Franke M Treatment of diarrhoea in human immunodeficiency virus-infected patients with immunoglobulins from bovine colostrum. *Clin Investig.* (1992) 70:588–94. 10.1007/BF00184800 1392428

[27] Ellens DJ de Leeuw PW Straver PJ. The detection of rotavirus specific antibody in colostrum and milk by ELISA. *Ann Rech Vet.* (1978) 9:337–42. 218492

[28] Warny M Fatimi A Bostwick EF Laine DC Lebel F LaMont JT Bovine immunoglobulin concentrate-clostridium difficile retains *C difficile* toxin neutralising activity after passage through the human stomach and small intestine. *Gut.* (1999) 44:212–7. 10.1136/gut.44.2.212 9895380 PMC1727384

[29] Jasion VS Burnett BP. Survival and digestibility of orally-administered immunoglobulin preparations containing IgG through the gastrointestinal tract in humans. *Nutr J.* (2015) 14:22. 10.1186/s12937-015-0010-7 25880525 PMC4355420

[30] Wong EB Mallet JF Duarte J Matar C Ritz BW. Bovine colostrum enhances natural killer cell activity and immune response in a mouse model of influenza infection and mediates intestinal immunity through toll-like receptors 2 and 4. *Nutr Res.* (2014) 34:318–25. 10.1016/j.nutres.2014.02.007 24774068

[31] Sponseller JK Steele JA Schmidt DJ Kim HB Beamer G Sun X Hyperimmune bovine colostrum as a novel therapy to combat *Clostridium difficile* infection. *J Infect Dis.* (2015) 211:1334–41. 10.1093/infdis/jiu605 25381448 PMC4447838

[32] Moreau MC Duval-Iflah Y Muller MC Raibaud P Vial M Gabilan JC [Effect of orally administered bovine lactoferrin and bovine IgG on the establishment of *Escherichia coli* in the digestive tract of gnotobiotic mice and human newborn infants]. *Ann Microbiol.* (1983) 134b:429–41. 6372577

[33] Kim JM Chung YS Jo HJ Lee NJ Kim MS Woo SH Identification of coronavirus isolated from a patient in Korea with COVID-19. *Osong Public Health Res Perspect.* (2020) 11:3–7. 10.24171/j.phrp.2020.11.1.02 32149036 PMC7045880

[34] Hashemi SA Golab Behbahan NG Bahrani S Mousavi SM Gholami A Ramakrishna S Ultra-sensitive viral glycoprotein detection nanoSystem toward accurate tracing SARS-CoV-2 in biological/non-biological media. *Biosensors Bioelectron.* (2021) 171:112731. 10.1016/j.bios.2020.112731 33075725 PMC7558249

[35] Le Bras A. Syrian hamsters as a small animal model for COVID-19 research. *Lab Anim.* (2020) 49:223. 10.1038/s41684-020-0614-1 32690933

[36] Imai M Iwatsuki-Horimoto K Hatta M Loeber S Halfmann PJ Nakajima N Syrian hamsters as a small animal model for SARS-CoV-2 infection and countermeasure development. *Proc Natl Acad Sci USA.* (2020) 117:16587–95. 10.1073/pnas.2009799117 32571934 PMC7368255

[37] Calder PC. Nutrition, immunity and COVID-19. *BMJ Nutr Prevent Health.* (2020) 3:74–92. 10.1136/bmjnph-2020-000085 33230497 PMC7295866

[38] Calder PC. Nutrition and immunity: lessons for COVID-19. *Nutr Diabetes.* (2021) 11:19. 10.1038/s41387-021-00165-0 PMC8223524 34168111

[39] Merino J Joshi AD Nguyen LH Leeming ER Mazidi M Drew DA Diet quality and risk and severity of COVID-19: a prospective cohort study. *Gut.* (2021) 70:2096–104. 10.1136/gutjnl-2021-325353 34489306 PMC8500931

[40] Singh S Diwaker A Singh BP Singh RK. Nutritional immunity, zinc sufficiency, and COVID-19 mortality in socially similar European populations. *Front Immunol.* (2021) 12:699389. 10.3389/fimmu.2021.699389 34603280 PMC8484327

[41] Parvathaneni V Gupta V. Utilizing drug repurposing against COVID-19 – efficacy, limitations, and challenges. *Life Sci.* (2020) 259:118275. 10.1016/j.lfs.2020.118275 32818545 PMC7430345

[42] Ehrlich P. Über Immunität durch Verebung Zeugung *Zeits Hygiene Infektion*. (1892) 12:183–203.

[43] Korhonen H Marnila P Gill HS. Bovine milk antibodies for health. *Br J Nutr.* (2000) 84 (Suppl. 1):S135–46. 10.1017/s0007114500002361 11242458

[44] Jawhara S. Can drinking microfiltered raw immune milk from cows immunized against SARS-CoV-2 provide short-term protection against COVID-19? *Front Immunol.* (2020) 11:1888. 10.3389/fimmu.2020.01888 32849647 PMC7399080

